# Supplementary material for: Coordinated calcium signalling in cochlear sensory and non‐sensory cells refines afferent innervation of outer hair cells
Source: EMBO J. 2019 Feb 25;38(9):e99839. doi: 10.15252/embj.201899839 (PMC6484507; doi:10.15252/embj.201899839)
Supplement: Supplementary file 1 — Appendix [file EMBJ-38-e99839-s001.docx]

**Coordinated calcium signalling in cochlear sensory and non-sensory cells refines afferent innervation of outer hair cells**

Federico Ceriani, Aenea Hendry, Jing-Yi Jeng, Stuart L. Johnson, Friederike Stephani, Jennifer Olt_,_ Matthew C. Holley, Fabio Mammano, Jutta Engel, Corné J. Kros, Dwayne D. Simmons, Walter Marcotti

**Appendix**

This document includes 9 Supplementary Figures and one Appendix Reference

**Supplementary Figure 1**

**

**

**Supplementary Figure 1. Spontaneous Ca^2+^ spikes in OHCs are Ca^2+^ dependent**

**A.** Relative fluo-4 fluorescence changes (Δ*F/F_0_*) before (left) during (middle) and after (right) the application of a Ca^2+^-free extracellular solution. Images were obtained as maximal back projections (500 frames, 16.5 s).

**B.** Representative *ΔF/F_0_* traces from 16 OHCs (from the images in panel *A*) during local application of the Ca^2+^-free extracellular solution with the picospritzer. Traces are computed as pixel averages of regions of interest centred on OHCs. Ca^2+^ spikes are evident in OHCs before the application of the Ca^2+^-free extracellular solution. The large Ca^2+^ transient at the onset of the Ca^2+^-free solution is most likely due to the increased open probability of the mechanoelectrical transducer channel (due to the removal of adaptation), and the consequent OHC depolarization (**Corns *et al*, 2018**). Note that the slow re-appearance of the Ca^2+^ spikes was due to the fact that the control solution containing 1.3 mM Ca^2+^ was bath perfused. A total of 5 recordings were performed from four cochleae (2 mice age P2). Recordings are at RT.

**B.** Maximum Δ*F/F_0_* changes before (black circles) and after (red circles) the extracellular application of Ca^2+^-free solution. Number of recordings is shown. *P* = 0.0134 (paired *t-*test).

**D.** Representative *ΔF/F0* traces from *Ca_V_1.3^-/-^* mouse (selected from the image in the right-hand panel) recorded at room temperature. Ca^2+^ spikes were never recorded in any of the OHCs from 15 recordings from 6 cochleae (3 mice) at P2. Calcium traces were computed as in **Fig 1C**.

**E.** Representative Δ*F/F_0_* traces from the same four P2 OHCs (colour coded) before (top panel) and during (bottom panel) the application of 60 µM ryanodine, which blocks the Ca^2+^ efflux from ryanodine-sensitive stores (**Meissner, 1986**). Note that ryanodine was applied for at least 30 minutes before re-imaging OHC activity. A total of 16 recordings were performed from 11 cochleae (7 mice age P2-P3). The estimated frequency of Ca^2+^ spikes (see Materials and Methods for details) was found to be similar between control OHCs (0.24 ± 0.02 Hz, *n* = 7 recordings) and those in the presence of ryanodine (0.22 ± 0.02 Hz, *P*<0.510, *t*-test). For both experimental conditions, recordings were made from 7 cochleae (7 mice). Recordings are at RT.

**Supplementary Figure 2**

**

**

**Supplementary Figure 2. The amplitude of Ca^2+^ waves does not affect OHC Ca^2+^-signals**

**A,C.** Average Spearman’s rank correlation coefficient (*r*_s_^avg^: see **Materials and Methods**) between the Ca^2+^ activity in wild-type (**A**) and *Cx30^-/-^* (**C**) OHCs as a function of the maximum *ΔF/F_0_* changes of spontaneous Ca^2+^ waves in the GER from the apical coil of P1-P2 mouse cochleae. Data were from the same OHCs analysed in **Fig 2F**.

**B,D.** Time integral of the fluorescence traces recorded from wild-type (**B**) and *Cx30^-/-^* (**D**) OHCs (see **Materials and Methods**) as a function of the maximum *ΔF/F_0_* changes of spontaneous Ca^2+^ waves in the GER from the apical coil of P1-P2 mouse cochleae.

The amplitude of the Ca^2+^ waves (Δ*F/F_0_*) was measured as a pixel average over the entire spread of the event.

**Supplementary Figure 3**

**

**

**Supplementary Figure 3. Calcium waves travel from the GER to the LER**

**A.** Simultaneous recordings of whole-cell voltage responses in a Deiters’ cell in the LER (black trace) and a spontaneous Ca^2+^ wave from the GER (*ΔF/F_0_*) of a wild-type P2 mouse. Note that although the Ca^2+^ signal originating in the GER precedes that recorded in the Deiters’ cells (**Fig 3D**), this in difficult to see in panel **A** because the time constant with which the electrical signal (Deiters’ cell: black) is recorded is much faster than that of the Ca^2+^ signal (GER, green). Scale bar: 20 μm.

**B.** Representative *ΔF/F_0_* traces (right) from the GER, Deiters’ cells and one OHC (from the left image) of a P1 *Ca_V_1.3^-/-^* mouse. Traces are computed as pixel averages of regions of interest. Note that OHCs do not show any Ca^2+^ signals due to absence of Ca^2+^ channels. These data provide evidence that repetitive Ca^2+^ signals in the GER can be mirrored in the Deiters’ cells.

Note that the Ca^2+^ signal from the GER (red) is integrated over the entire area of the Ca^2+^ wave, and therefore inevitably includes signals propagating among non-sensory cells in all directions (e.g. orthogonal to and along the coil of the cochlea). The Deiters’ cell Ca^2+^ signal was from either a single cell (blue), or integrated across a few cells (green). The different analysis used for the GER (red) and single Deiters’ cell trace (blue) give the impression that the latter trace begins to return to the baseline fluorescence before that from the GER.

**Supplementary Figure 4**

**

**

**Supplementary Figure 4. OHC responses in the presence and absence of Deiters’ cells.**

**A,B.** Left panels: diagram showing a cross-section of an early postnatal organ of Corti with the tip of the patch pipette attached to the OHC. The recordings are performed in the presence (**A**) or absence (**B**) of the Deiters’ cells. Right panels show DIC images of the cochlea at the level of the OHCs (green squares) and non-sensory Deiters’ cells (blue squares).

**C,D**. Typical current responses from P2 apical-coil OHCs recorded from cochleae in which Deiters’ cells were retained (**C**) or removed (**D**), as shown in panels (**A**) and (**B**), respectively. Outward K^+^ currents were elicited by using depolarizing voltage steps (10 mV increments) from –84 mV to the various test potentials shown by some of the traces.

**E**. Average size of the total outward K^+^ current measured at 0 mV (*I*_K_: left panel), OHC membrane capacitance (*C*_m_: middle panel) and resting membrane potential (*V*_m_: right panel) in the presence (black circle) and absence (red circles) of the Deiters’s cells. Number of OHCs investigated is shown above the columns. Values were not significantly different: *I*_K_: *P* = 0.9032; *C*_m_: *P* = 0.5915; *V*_m_: *P* = 0.6358, *t-*test.

**F,G**. Spontaneous action potentials recorded from P2 apical-coil OHCs in the presence (**F**) and absence (**G**) of the Deiters’ cells. Recordings were performed using physiological 1.3 mM extracellular Ca^2+^ (room temperature).

**H**. Representative *ΔF/F_0_* traces from 4 apical OHCs of a P1 wild-type mouse recorded at room temperature (recordings as in **Fig 1C**) but in the absence of the Deiters’ cells.

**I**. Representative *ΔF/F_0_* traces from 9 apical OHCs of P1 wild-type mice during the application of 100 μM ATP without the removal of Deiters’ cells.

**Supplementary Figure 5**

**

**

**Supplementary Figure 5. Large spontaneous Ca^2+^ signalling events from the GER cause graded Ca^2+^ elevation in OHCs**

**A.** Representative images showing the initiation of a spontaneous large Ca^2+^ wave (top) and its full extent (bottom). The red area in the top panel is the region used to measure Ca^2+^ signals at the point where the Ca^2+^ wave started (GER in panel **B**). The blue ROIs are those used to measure OHC Ca^2+^ signalling in panel **B**. Recordings were performed at RT from wild-type P2 mice.

**B.** Representative *ΔF/F_0_* traces from the GER (red traces) and OHCs (blue traces). Note that the 6 OHCs traces named “OHC1” are those closer to the large Ca^2+^ wave starting position in the GER (top panel **A**), while the 7 OHC traces named “OHC2” are those far away from the Ca^2+^ wave starting position.

**Supplementary Figure 6**

**

**

**Supplementary Figure 6. ATP-induced responses in OHCs from wild-type mice**

Representative *ΔF/F_0_* traces from 9 apical OHCs of P1 wild-type (**A**, top) and *Ca_V_1.3^-/-^* (**B**, top) mice during the application of 100 μM ATP. Data analysis as in **Fig 1C**. Recordings are at RT. Voltage responses in P1 OHCs from wild-type (**A**, bottom) and *Ca_V_1.3^-/-^* (**B**, bottom) mice during the application of 100 μM extracellular ATP. Note that in the absence of Ca^2+^ channels, ATP induces a transient voltage change that is most likely due to current through the P2X receptors. For the above experiments one or two rows of Deiters’ cells were removed. Recordings are at RT.

**Supplementary Figure 7**

**

**

**Supplementary Figure 7. The absence of connexins does not affect the function of immature OHCs**

**A,B.** Typical current responses from P2 apical-coil OHCs recorded from wild-type (**A**) and *Cx30^-/-^* (**B**) mice. Outward K^+^ currents were elicited by using depolarizing voltage steps (10 mV increments) from –84 mV to the various test potentials shown by some of the traces.

**C**. Average size of the total outward K^+^ current measured at 0 mV (*I*_K_: left panel), OHC membrane capacitance (*C*_m_: middle panel) and resting membrane potential (*V*_m_: right panel) in the presence (black circle) and absence (red circles) of the Deiters’ cells in both genotypes. Number of OHCs investigated is shown above the columns. Values were not significantly different: *I*_K_: *P* = 0.4215; *C*_m_: *P* = 0.2438; *V*_m_: *P* = 0.2187, *t-*test.

**D,E**. Spontaneous action potentials recorded from P2 apical-coil OHCs in wild-type and *Cx30^-/-^* mice, respectively. Recordings were performed using physiological 1.3 mM extracellular Ca^2+^ (room temperature).

**Supplementary Figure 8**

**

**

**Supplementary Figure 8. The number of ribbon synapses is not affected in *Cx30^-/-^* IHCs**

**A-D.** Maximum intensity projections of confocal z-stack images that were taken from apical coil IHCs at P4 and P10 of wild-type (**A** and **C**) and *Cx30^-/-^* (**B** and **D**) mice. Immunostaining for ribbon synapses (CtBP2) is shown in red; Myo7a (blue) was used as the hair cell marker.

**E.** Number of ribbons (CtBP2 puncta) in wild-type and *Cx30^-/-^* IHCs at P4 and P10. Values are mean ± SEM. Number of IHCs analysed is shown above each average data point; 4 mice were used for each experimental condition. Scale bars 10 μm.

**Supplementary Figure 9**

**

**

**Supplementary Figure 9. OHCs from *Cav1.3^-/-^* mice are normal**

**A,B.** DIC images showing a region of the mouse cochlear apical coil showing the presence of OHCs at P11 for wild-type (**A**) and *Ca_V_1.3^-/-^* (**B**) mice. Note that the hair bundle of the first row of OHCs is visible.

The number of OHCs present in a 200 μm region from the apical cochlear coil of wild-type mice (75 ± 1.7, *n* = 4, P11 mice) was not significantly different to that present in *Ca_V_1.3^-/-^* mice (79 ± 1.5, *n* = 5, P11 mice, *P*=0.1205).

The cell membrane capacitance (*C*_m_), which provides an indication of the cells surface, and the resting membrane potential (*V*_m_) were also no significantly different between wild-type (*C*_m_: 7.4 ± 0.2 pF, *n* = 9; *V*_m_: -65.7 ± 2.1 mV, *n* = 8, P11 mice) and *Ca_V_1.3^-/-^* (*C*_m_: 6.9 ± 0.2 pF, *n* = 2, *P*=0.2936; *V*_m_: -60.7 ± 0.9 mV, *n* = 4, *P*=0.1381, P11 mice) OHCs.

**Appendix Reference**

Meissner G (1986) Ryanodine activation and inhibition of the Ca^2+^ release channel of sarcoplasmic reticulum. *J Biol Chem* 261: 6300 – 6306
