# Supplementary material for: Coordinated calcium signalling in cochlear sensory and non‐sensory cells refines afferent innervation of outer hair cells
Source: EMBO J. 2019 Feb 25;38(9):e99839. doi: 10.15252/embj.201899839 (PMC6484507; doi:10.15252/embj.201899839)
Supplement: Supplementary file 2 — Movie EV1 [file EMBJ-38-e99839-s002.zip › Movie_EV1.docx]

**Movie EV1**


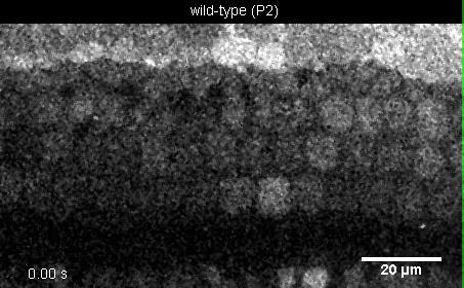


Recording of spontaneous Ca^2+^-dependent action potential activity at body temperature from P2 apical coil OHCs loaded with Fluo-4 AM.
