# Supplementary material for: Coordinated calcium signalling in cochlear sensory and non‐sensory cells refines afferent innervation of outer hair cells
Source: EMBO J. 2019 Feb 25;38(9):e99839. doi: 10.15252/embj.201899839 (PMC6484507; doi:10.15252/embj.201899839)
Supplement: Supplementary file 3 — Movie EV2 [file EMBJ-38-e99839-s003.zip › Movie_EV2.docx]

**Movie EV2**


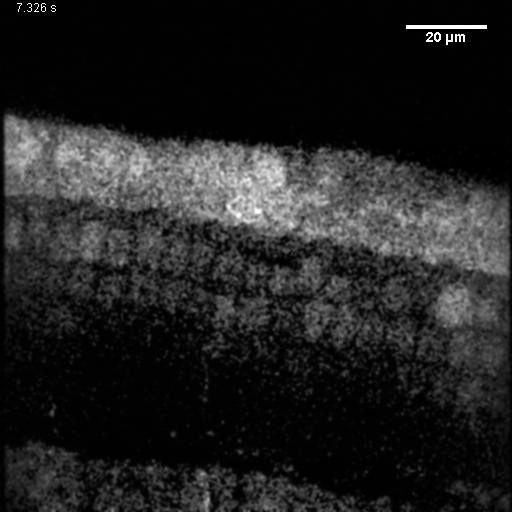


Recording of spontaneous activity in immature OHCs while perfusing a Ca^2+^-free extracellular solution, which prevents spontaneous spiking.
