# Supplementary material for: Coordinated calcium signalling in cochlear sensory and non‐sensory cells refines afferent innervation of outer hair cells
Source: EMBO J. 2019 Feb 25;38(9):e99839. doi: 10.15252/embj.201899839 (PMC6484507; doi:10.15252/embj.201899839)
Supplement: Supplementary file 4 — Movie EV3 [file EMBJ-38-e99839-s004.zip › Movie_EV3.docx]

**Movie EV3**

**
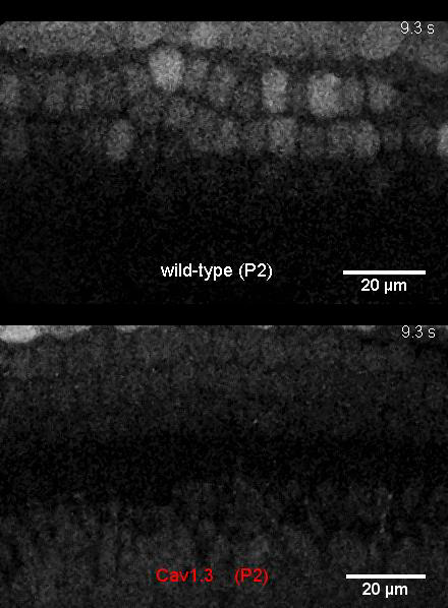
**

Comparison between two representative Ca^2+^ imaging recordings from wild-type (top) and *Cav1.3^-/-^* (bottom) OHCs loaded with Fluo-4 AM. Note the absence of Ca^2+^ spikes in the *Cav1.3^-/-^* preparation.
