# Supplementary material for: Coordinated calcium signalling in cochlear sensory and non‐sensory cells refines afferent innervation of outer hair cells
Source: EMBO J. 2019 Feb 25;38(9):e99839. doi: 10.15252/embj.201899839 (PMC6484507; doi:10.15252/embj.201899839)
Supplement: Supplementary file 5 — Movie EV4 [file EMBJ-38-e99839-s005.zip › Movie_EV4.docx]

**Movie EV4**

**
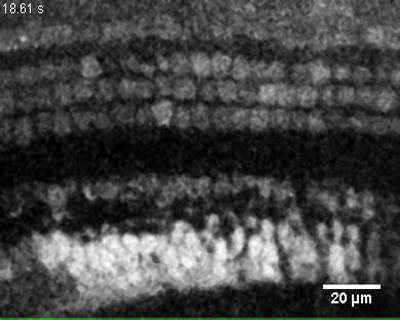
**

Recording of spontaneous activity in immature OHCs and spontaneous Ca^2+^ waves in the GER. Note the simultaneous increase in OHC firing during a large Ca^2+^ wave activity in the GER.
