# Supplementary material for: Coordinated calcium signalling in cochlear sensory and non‐sensory cells refines afferent innervation of outer hair cells
Source: EMBO J. 2019 Feb 25;38(9):e99839. doi: 10.15252/embj.201899839 (PMC6484507; doi:10.15252/embj.201899839)
Supplement: Supplementary file 6 — Movie EV5 [file EMBJ-38-e99839-s006.zip › Movie_EV5.docx]

**Movie EV5**

**
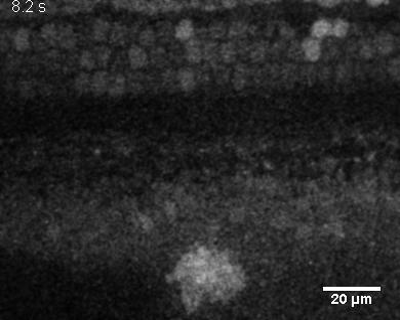
**

Recording of spontaneous Ca^2+^ signals in immature OHCs and spontaneous Ca^2+^ waves in the GER. Small Ca^2+^ waves fail to trigger an increase in firing in nearby OHCs.
