# Supplementary figures and images for: Coordinated calcium signalling in cochlear sensory and non‐sensory cells refines afferent innervation of outer hair cells
Source: EMBO J. 2019 Feb 25;38(9):e99839. doi: 10.15252/embj.201899839 (PMC6484507; doi:10.15252/embj.201899839)

**Movie EV6**

**
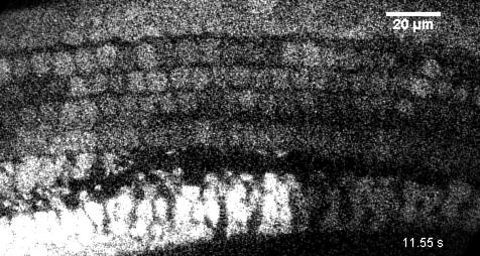
**

Recording of Ca2+ activity in immature OHCs and photodamage-induced Ca2+ waves in the GER.

Supplement: Supplementary file 7 — Movie EV6 [file EMBJ-38-e99839-s007.zip › Movie_EV6.docx]
