# Supplementary material for: Coordinated calcium signalling in cochlear sensory and non‐sensory cells refines afferent innervation of outer hair cells
Source: EMBO J. 2019 Feb 25;38(9):e99839. doi: 10.15252/embj.201899839 (PMC6484507; doi:10.15252/embj.201899839)
Supplement: Supplementary file 8 — Movie EV7 [file EMBJ-38-e99839-s008.zip › Movie_EV7.docx]

**Movie EV7**

**
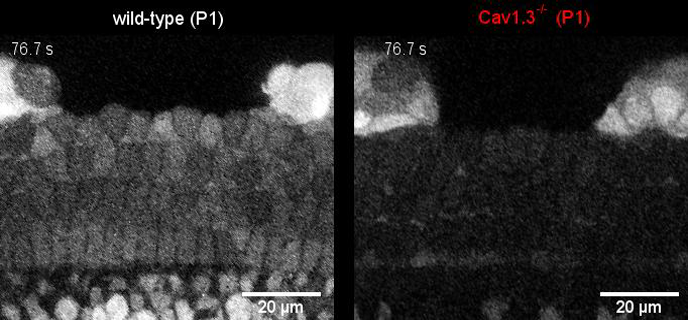
**

Comparison between two representative Ca^2+^ imaging recordings from wild-type (left) and *Ca_V_1.3^-/-^* (right) OHCs during the application of 100 µM ATP. Deiters’ cells intercalating OHCs are clearly visible in the *Ca_V_1.3^-/-^* recording due to the absence of response in OHCs.
