# Supplementary material for: Coordinated calcium signalling in cochlear sensory and non‐sensory cells refines afferent innervation of outer hair cells
Source: EMBO J. 2019 Feb 25;38(9):e99839. doi: 10.15252/embj.201899839 (PMC6484507; doi:10.15252/embj.201899839)
Supplement: Supplementary file 9 — Movie EV8 [file EMBJ-38-e99839-s009.zip › Movie_EV8.docx]

**Movie EV8**

**
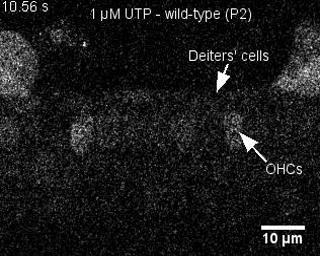
**

Representative recording showing Ca^2+^ responses in OHCs and non-sensory cells induced by application of 1 µM UTP.
